# Preparation and Applicability Evaluation of High-Temperature-Resistant, Breakable Resin–Gel Plugging Agent

**DOI:** 10.3390/gels12020164

**Published:** 2026-02-13

**Authors:** Tao Wang, Jinzhi Zhu, Yingrui Bai, Yanming Yin, Qisheng Jiang, Zhangkun Ren, Jingbin Yang

**Affiliations:** 1 R&D Center for Ultra Deep Complex Reservior Exploration and Development, China National Petroleum Corporation, Korla 841000, China; wangtao953@126.com (T.W.); zhujz147@163.com (J.Z.); jiangq4321@126.com (Q.J.); 2 Engineering Research Center for Ultra-Deep Complex Reservoir Exploration and Development, Xinjiang Uygur Autonomous Region, Korla 841000, China; 3 Tarim Oilfield Branch, China National Petroleum Corporation, Korla 841000, China; 4 Xinjiang Key Laboratory of Ultra-Deep Oil and Gas, Korla 841000, China; 5 School of Petroleum Engineering, China University of Petroleum (East China), Qingdao 266580, China; cczurzk@163.com (Z.R.); yangjingbin2018@163.com (J.Y.); 6 Korla Branch, Bohai Drilling Engineering Company Limited, China National Petroleum Corporation, Korla 841000, China; yanmingyin43@126.com

**Keywords:** high-temperature-resistant resin–gel, fractured-solids reservoir, controlled degradation, high-temperature resistance, gas flow control

## Abstract

This study addresses the challenge of high-temperature gas channeling in injection–production wells of karst-fractured reservoirs by developing a high-temperature-resistant resin–gel plugging system capable of withstanding up to 150 °C. The system employs an AMPS/NVP copolymer (molar ratio 3:1) as the polymer matrix, reinforced with phenolic resin to enhance the crosslinked network. Additionally, a polyamide microcapsule was utilized to encapsulate the gel breaker, enabling controlled release. The optimized formulation consists of 0.5% NEP, 0.5% DEP, 0.6% HMTA, 0.3% catechol, and 25% resin curing agent. Experimental results demonstrate that the system exhibits excellent stability at 150 °C, with a G′ ≥ 125 Pa and compressive strength > 18 MPa. It also displays strong contamination resistance, showing a viscosity reduction of <9.7% and a storage modulus retention rate > 87% after mixing with drilling fluid. Furthermore, the gel-breaking performance is controllable, achieving a gel-breaking rate ≥ 99.7% within 21 days. Under high-temperature and high-pressure conditions (150 °C), the system demonstrates a plugging efficiency > 92% for simulated fractures with widths ranging from 0.1 to 2 mm. This technology effectively suppresses gas channeling in complex high-temperature formations, making it suitable for gas injection wells in karst-fractured reservoirs. It also holds promise for extension to shale gas wells and geothermal reservoir sealing applications.

## 1. Introduction

Chemical plugging technology plays a critical role in the development of heterogeneous oil and gas reservoirs. Its core mechanism lies in selectively blocking high-permeability channels to redirect fluid flow [1]. Conventional plugging systems mainly include polymer gels, particulate plugging agents, and foam-based systems. Among them, polymer gels are widely applied due to their tunable rheological properties and controllable gelation kinetics [2,3,4]. From a rheological perspective, a gel is defined as a viscoelastic solid material characterized by a storage modulus (G′) that exceeds the loss modulus (G″) over a broad frequency range, indicating the formation of a percolated network structure. Key rheological measurements for gel characterization include amplitude sweeps, frequency sweeps, and recovery tests, which allow for the evaluation of network formation, mechanical strength, and thermal stability of gel systems. Polyacrylamide-based gels are among the most commonly used gels; however, they are prone to molecular chain scission and structural collapse under high-temperature (>90 °C) or high-salinity (>20,000 ppm) conditions [5,6]. This degradation mainly stems from the hydrolytic sensitivity of amide groups and metal-ion-induced aggregation–precipitation [7]. Particulate plugging agents (e.g., pre-crosslinked gel particles and nano-silica) exhibit excellent thermal and salt resistance due to their rigid structures. However, the challenge of achieving precise particle size compatibility with reservoir pore structures remains unresolved [8,9]. Foam systems achieve dynamic plugging by reducing fluid loss via gas–liquid phase interactions, yet they face technical obstacles such as phase separation and thermal degradation of surfactants [10]. Therefore, the development of multifunctional gel systems with strong temperature adaptability and excellent structural stability has become a focal point of current research.

In recent years, the development of high-temperature-resistant gel systems has attracted significant attention in response to the oil industry’s trend of extending exploration and production into deep (>4500 m) and ultra-deep (>8000 m) reservoirs [11,12]. Deep reservoir formations often exhibit temperatures exceeding 150 °C and salinities up to 300,000 mg/L, posing significant challenges to the thermal and salt stability of polymer gels [13]. Current strategies to enhance gel thermal stability primarily include: (i) optimizing the polymer backbone by incorporating thermally stable groups such as sulfonic acid and cyclic structures, (ii) developing organic–inorganic hybrid crosslinking systems to construct dual-network architectures, (iii) introducing nanomaterials to fabricate hybrid reinforced gels [14,15,16]. Liu et al. [17] developed a lignin-based hydrogel for leakage control, which exploits the self-crosslinking ability of lignin under elevated temperatures. This approach reduced the dosage of chemical crosslinkers, enabling tunable gelation times while improving the mechanical strength. The hydrogel also exhibited excellent thermal resistance, showing minimal mass loss during thermal decomposition at temperatures below 220 °C, making it suitable for high-temperature applications. Bai et al. [18] synthesized a P(AM-co-AMPS)/SADN gel via aqueous polymerization. The gel featured a dual-network structure—a hydrophobically associated crosslinked network between acrylamide (AM) and lauryl methacrylate (LMA)—and an ionic network formed through interactions between sodium alginate and Ca^2+^ ions. To further enhance thermal resistance, 2-acrylamido-2-methylpropanesulfonic acid (AMPS) was incorporated into the formulation, endowing the gel with superior temperature stability and a porous structure.

The selection of crosslinkers plays a critical role in determining the high-temperature stability of gels. Currently, mainstream crosslinkers can be categorized into three major types: phenolic compounds (resorcinol), metal ions (Cr^3+^, Al^3+^), and organic macromolecules (polyethyleneimine) [19,20,21]. Phenolic crosslinkers form stable covalent bonds (such as ether linkages) with polymers, offering strong thermal stability, while their benzene ring structures further enhance network rigidity. Metal-ion-based crosslinkers rely on coordination bonds, which are susceptible to hydrolysis and cleavage under high-temperature and high-salinity conditions, resulting in relatively poor stability. Organic polymer crosslinkers, on the other hand, combine covalent crosslinking with physical entanglement, creating interpenetrating networks that are resistant to both temperature and creep deformation. Consequently, phenolic and polymeric crosslinkers are more suitable for high-temperature environments, while metal-ion systems are typically used under moderate to low temperatures or as auxiliary crosslinking components [22]. Lu et al. [23] developed a novel nano-graphite hybrid crosslinked gel using polyacrylamide (PAM) as the polymer matrix, with hexamethylenetetramine (HMTA), hydroquinone (HQ), and modified nano-graphite (MG) as crosslinkers. The gelation time of this hybrid gel system was 4 h, and the maximum tolerable temperature reached 154.9 °C. Furthermore, the incorporation of MG more than doubled the gel’s storage modulus (G′) and loss modulus (G″), indicating enhanced viscoelastic properties. The presence of MG significantly increased the network strength and crosslinking density of the hybrid gel, thereby improving its thermal stability, heat resistance, and salt tolerance. Wei et al. [24] successfully synthesized a high-temperature-resistant polymer gel (T-PPG) by replacing the conventional crosslinker N,N′-methylenebisacrylamide (MBA) in modified PPG (M-PPG) with triamine (TAA) and systematically evaluated its performance. Core flooding experiments revealed that T-PPGs retained considerable plugging performance even after aging at 140 °C for 60 h. Compared with M-PPGs, the T-PPGs demonstrated superior thermal stability. Conventional phenolic crosslinking systems still face challenges, such as high network brittleness and poor degradation controllability, under extreme high-temperature conditions. These limitations can be effectively addressed by constructing organic–inorganic hybrid structures or multiple dynamic crosslinked networks to further enhance the gel architecture. For instance, the introduction of reversible covalent bonds or rigid aromatic structural units facilitates the formation of thermally reinforced dual-crosslinked and hierarchical networks, which significantly improves the long-term stability and mechanical performance of gels under harsh conditions, including high temperatures and high salinity.

The degradation mechanisms of gel stability in high-salinity environments primarily involve two aspects: (1) the ionic shielding effect reduces the hydrophilicity of polymer chains, leading to network shrinkage, and (2) divalent/trivalent ions form chelation complexes with carboxyl groups on the polymer backbone, disrupting the crosslinked network [25,26]. To address these challenges, researchers have proposed strategies focused on molecular structural optimization. These include using low-molecular-weight polymers to reduce chain entanglement and improve chain extensibility in saline environments, constructing topologically advanced architectures such as star-shaped or comb-like polymers to enhance intermolecular interactions and introducing zwitterionic functional groups to neutralize charge screening effects [27,28,29]. Guo et al. [30] investigated the effects of crosslinker concentration on gel stability and gelation time. The presence of AMPS in the crosslinked polymer network was found to govern the gel’s resistance to high temperature and salinity. Utilizing AM-AMPS copolymers with high AMPS content, along with tuning the crosslinker concentration, effectively suppressed intramolecular catalytic hydrolysis of AMPS units, thereby improving gel stability. These findings provide valuable insights for designing and developing conformance control gels suitable for high-temperature, high-salinity reservoirs. Liu et al. [31] focused on a novel polymer particle—soft microgels (SMGs)—and evaluated their physical and chemical properties, as well as their performance in profile control and oil displacement, under high-salinity conditions (200,000 mg/L). The sub-millimeter-scale SMGs exhibited excellent swelling capacity, rheological behavior, and thermal stability. Remarkably, the SMGs remained structurally stable even after aging for six months under high-temperature, high-salinity conditions, without undergoing hydration or hydrolysis, as commonly observed in conventional polymers.

Although certain gel plugging systems have been implemented in field applications, they still face issues such as insufficient strength and poor high-temperature stability, which hinder their ability to meet on-site operational requirements. Based on dual-crosslinked network design and microcapsule-controlled gel-breaking, this study developed a novel high-temperature-resistant resin–gel plugging system. By optimizing the concentrations of key components, including high-temperature-resistant crosslinkable polymers and crosslinkers, the formulation of the high-temperature chemical plugging agent was determined. Furthermore, molecular structure characterization was performed to establish a thermally stable and breakable chemical plugging system. Field-specific performance metrics were investigated, including the system’s anti-contamination capability and its adaptability to high-density (weighted) formulations. Under high-temperature conditions (130 °C and 150 °C), after aging for 3 and 9 days respectively, high-temperature and high-pressure (HTHP) fluid loss showed minimal sensitivity to pressure variations. Physical simulation experiments were conducted to evaluate the gas-blocking performance of the system. These experiments confirmed the system’s effective anti-gas channeling capability under high-temperature conditions. Under both ambient temperature and high-temperature conditions of 150 °C, the gel-breaking rate reached up to 99.7% within 24 h. An evaluation methodology for high-temperature-resistant chemical plugging systems was established, providing crucial technical support for the efficient development of deep oil and gas reservoirs.

## 2. Results and Discussion

### 2.1. Construction of High-Temperature-Resistant Resin–Gel Plugging System

The thixotropic resin–gel plugging system constructed in this study consists of three components: polymer, organic phenolic resin crosslinking system, and urea–formaldehyde resin curing agent. The study mainly investigates the effects of factors such as polymer concentration, phenolic crosslinking agent, aldehyde crosslinking agent, and resin curing agent on the gelation performance of the system. Based on experimental results, the optimal composition and concentration of the system are selected to construct a high-temperature-resistant resin–gel plugging system.

#### 2.1.1. Preferred Polymer Concentration 

In the field of oilfield chemistry, the hydrolysis degree of polyacrylamide exhibits a significantly positive correlation with the carboxyl group (–COOH) content in its molecular structure. As the hydrolysis degree of the polymer increases, the proportion of carboxyl functional groups on its molecular chain also increases. This structural characteristic has an important impact on the crosslinking reaction: in organic crosslinking systems (such as phenolic resin crosslinkers and other polymer-based crosslinkers), the reaction mechanism is notably different, and the crosslinking sites mainly target the amide groups (–CONH_2_) in the polyacrylamide molecules [32]. To ensure sufficient crosslinking sites for the formation of a high-strength gel, the polyacrylamide (PAM) polymer employed should maintain a relatively high amide group content, which typically requires controlling its degree of hydrolysis within a low range of 10–30%. Within this range, the amide group concentration remains adequate and relatively stable, meaning that minor variations in hydrolysis degree have limited influence on the ultimate gelation performance. However, an excessively low degree of hydrolysis can significantly impair the hydration capacity of the polymer, leading to a sharp decline in dissolution rate, prolonged gelation time, and consequently, reduced operational efficiency. Therefore, an ideal polymer must balance the retention of sufficient amide groups for crosslinking with the presence of an appropriate amount of carboxyl groups to ensure satisfactory solubility. Based on the above mechanistic study and optimization analysis, two representative polymers, NEP and DEP, are selected for this study.

**Figure 1:**
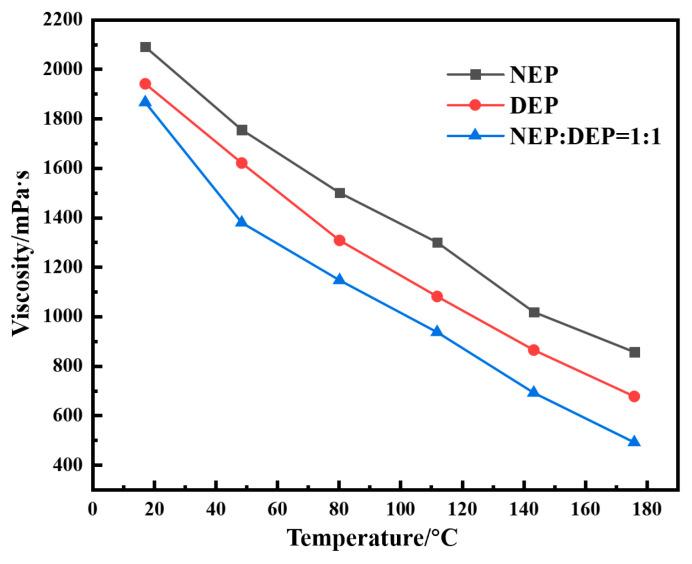
Comparison of the viscosity–temperature curves of single polymers and blended polymers at a mass fraction of 1%.

**Figure 2:**
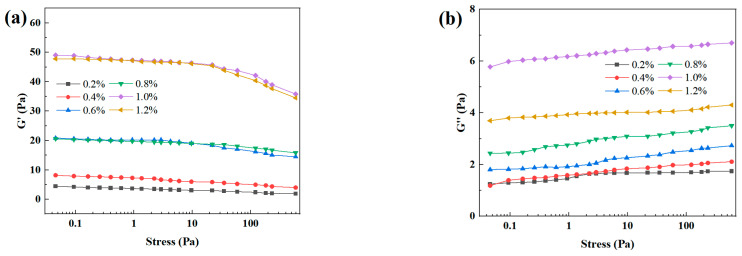
Effect of different polymer concentrations on the resin–gel. (**a**) Effect of different polymer concentrations on the energy storage modulus of the resin–gel. (**b**) Effect of different polymer concentrations on the loss modulus of the resin–gel.

In response to the harsh conditions of high temperature and salinity in deep formations, this study utilizes a blend of NEP and DEP polymers at a 1:1 mass ratio to investigate the influence of the blend ratio on the system’s viscosity–temperature behavior. Experimental results (Table 1) indicate that the viscosity of the blended system decreases monotonically with increasing temperature (80–180 °C), which is consistent with the viscosity–temperature behavior of individual polymers. This is attributed to enhanced thermal motion of polymer chains at elevated temperatures, weakening intermolecular interactions. Notably, under the same temperature conditions, the viscosity of the blended system significantly increases with the polymer mass fraction. Comparative analysis (Figure 1) shows that the viscosity values of the blended system are superior to those of the individual polymer systems at the same temperature. This improvement is likely due to the synergistic interaction between the NEP and DEP molecular chains, which enhances the molecular entanglement density and forms a more compact three-dimensional network structure. This synergistic effect not only retains the temperature and salt resistance characteristics of the individual components but also further enhances the overall viscosity performance of the system, offering a new technological approach for the optimization of chemically enhanced oil recovery systems in high-temperature, high-salinity reservoirs. In addition, Figure 2 compares the influence of different polymer concentrations on the gelation performance of the resin–gel system. As illustrated in Figure 2, within the entire tested concentration range, G′ consistently and significantly exceeds G″, indicating the formation of a typical elastic gel network structure. As the polymer concentration gradually increases from 0.2% to 1.0%, both G′ and G″ show an upward trend, which can be attributed to the increased crosslinking sites and enhanced network density resulting from the higher polymer chain concentration. However, when the concentration is further raised to 1.2%, both G′ and G″ exhibit a decrease. This decline may be related to the overly dense arrangement of polymer chains, which can lead to uneven distribution of crosslinking agents within the system. Such localized variations in crosslinking density are prone to creating structural defects in the network, ultimately compromising the overall mechanical performance of the gel.

This study systematically evaluated the gelation performance of a 1.0% total concentration NEP/DEP blended polymer system with phenolic resin crosslinkers under 180 °C high-temperature conditions. The experimental results (Table 2) show that when the two polymers are blended in a 1:1 mass ratio, the system exhibits optimal crosslinking performance, with the gel strength reaching the H-level standard. This synergistic effect arises from the complementary actions of the high-temperature functional groups (such as the sulfonic groups in AMPS and the rigid cyclic structures in NVP) on the molecular chains of NEP and DEP: on one hand, the inter-chain entanglement between different molecules enhances the spatial crosslinking density of the gel network. On the other hand, the thermally stable groups in DEP effectively suppress the hydrolysis of amide groups at high temperatures, thereby significantly improving the compactness and thermal stability of the gel framework. This blended system provides a reliable chemical solution for enhanced oil recovery operations in ultra-high-temperature reservoirs.

#### 2.1.2. Preferred Crosslinker Concentration

The study systematically evaluated the effect mechanism of hexamethylenetetramine (HMTA) as an aldehyde crosslinker and resorcinol as a phenolic crosslinker on the gelation performance of polymer gel systems.

**Figure 3:**
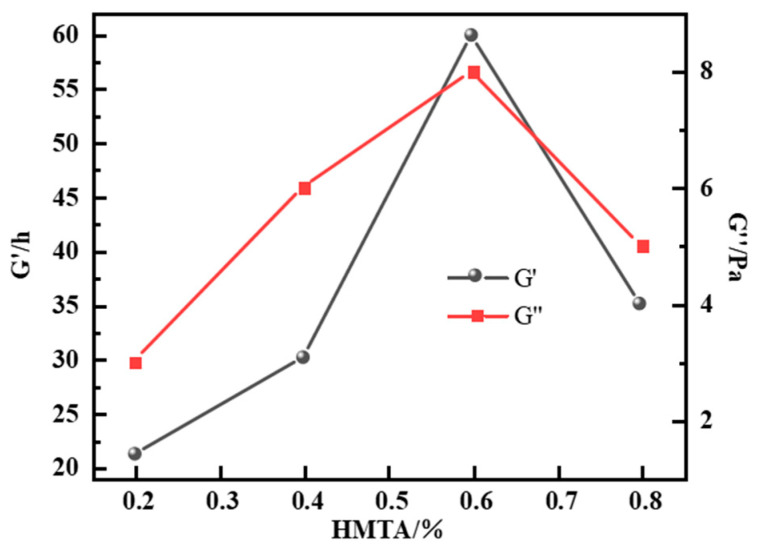
Effect of HMTA concentration on energy storage modulus of resin–gel.

The study found that HMTA decomposes at an appropriate rate under high-temperature conditions, generating formaldehyde, which can form a stable crosslinked network structure before the polymer molecular chains break. When the total polymer concentration is maintained at 1.0%, HMTA concentrations ranging from 0.2% to 0.8% can form gel systems, but the performance shows significant concentration dependence. As shown in Figure 3, as the HMTA concentration increases from 0.2% to 0.6%, the gel system exhibits optimal rheological properties at the concentration of 0.6%, with the G′ and G″ reaching 60 Pa and 8 Pa, respectively, indicating the formation of a three-dimensional network structure with ideal viscoelastic properties. This improvement results from a moderate crosslinking density between formaldehyde (from HMTA decomposition) and polymer chains. Notably, when the HMTA concentration exceeds 0.6%, a noticeable “over-crosslinking” phenomenon arises: the crosslinking reaction rate accelerates excessively, resulting in an uneven network structure. Additionally, some molecular chains are “locked” in the crosslinked state, thereby reducing the gel’s mechanical strength. This phenomenon is confirmed by the simultaneous decrease in both the storage modulus and loss modulus. Therefore, considering both gelation time and mechanical properties, 0.6% is determined to be the optimal concentration of HMTA, at which the formed gel system exhibits excellent gelation kinetics and mechanical stability, fully meeting the technical requirements for enhanced oil recovery operations in high-temperature reservoirs.

**Figure 4:**
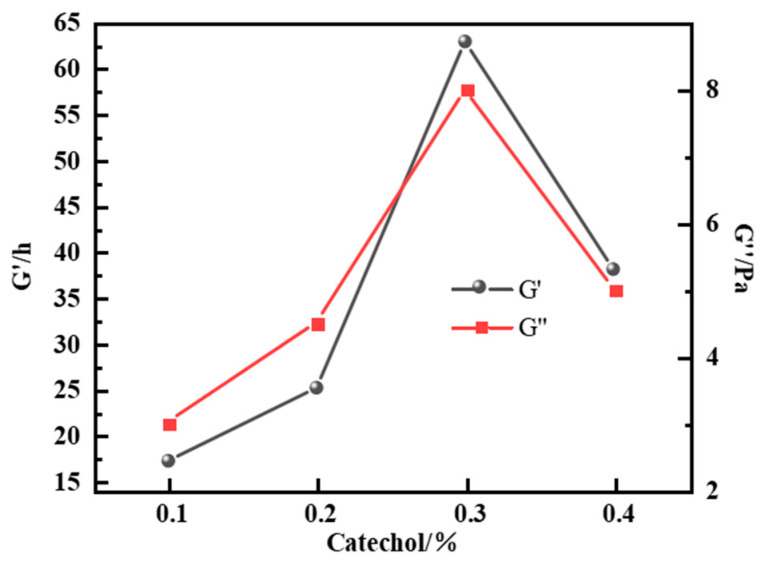
Effect of catechol concentration on the energy storage modulus of the resin–gel.

As shown in Figure 4, when catechol was employed as the phenolic crosslinker, the gel system exhibited excellent performance at 150 °C. At a catechol concentration of 0.3%, the G′ and G″ of the gel reached their maximum values of 63 Pa and 9 Pa, respectively, while the wall-hanging performance was also optimal. Although the gelation time shortened with increasing catechol concentration, both the G′ and G″ showed a decreasing trend when the concentration exceeded 0.3% due to an over-crosslinking effect, which is consistent with the behavior observed for aldehyde-based crosslinkers. This indicates that 0.3% is the optimal concentration for catechol. The selected system formed a stable crosslinked network structure at high temperature, providing a reliable gel system for plugging operations in high-temperature formations.

#### 2.1.3. Optimization of Resin Curing Agent Concentration

**Figure 5:**
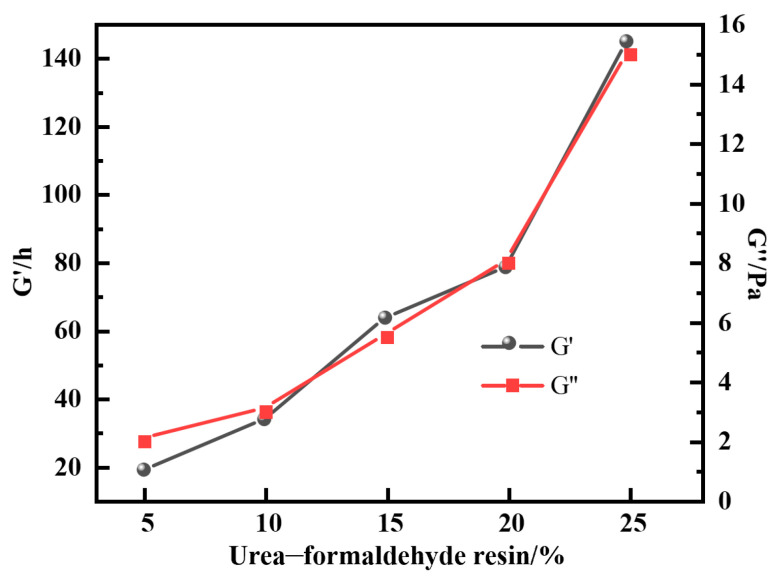
Effect of urea–formaldehyde resin concentration on energy storage modulus of resin–gel.

In this study, by introducing a urea–formaldehyde resin curing agent into the gel plugging agent system, the stability of the system under high-temperature conditions was significantly improved. The effect of urea–formaldehyde resin concentration (5–25%) on the gel properties was systematically investigated at 150 °C using a system of 1.0% polymer, 0.6% hexamethylenetetetramine and 0.3% catechol. The results showed that active functional groups in urea–formaldehyde resin formed a three-dimensional network via self-polymerization. This created a hybrid system with the polymer gel network, effectively enhancing the gel’s temperature resistance and mechanical strength. As the resin concentration increased to 25%, the energy storage modulus and loss modulus were elevated to 142 Pa and 15 Pa, respectively (Figure 5). The hybridized structure significantly improved the pressure-bearing sealing ability of the gel for large crack leakage channels. When the resin concentration exceeded 25%, gel viscoelasticity deteriorated and dehydration occurred. This is attributed to excessive crosslinking, which overly rigidifies the network and compresses the pore structure, thereby expelling free water from the system [21]. Consequently, a concentration of 25% was identified as the optimal level. This optimized formulation provides a reliable technical solution for plugging operations in high-temperature reservoirs.

#### 2.1.4. Construction of High-Temperature-Resistant Gel Tampering System

**Figure 6:**
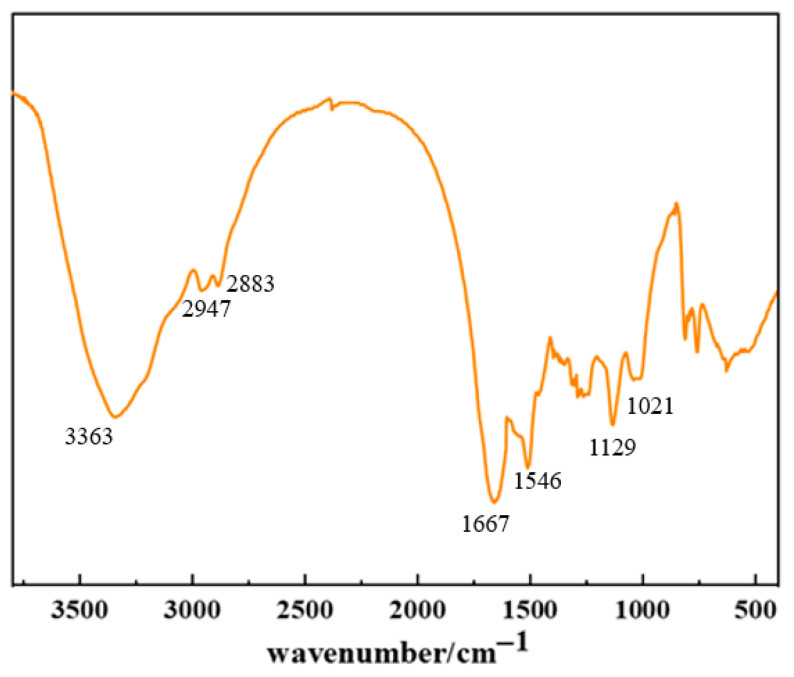
Infrared structural characterization of resin–gel.

The optimized formulation of the resin–gel plugging system was determined as 0.5% NEP + 0.5% DEP + 0.6% HMTA + 0.3% catechol + 25% resin curing agent. The structure of the system was characterized by Fourier transform infrared (FTIR) spectroscopy, and the results are presented in Figure 6. The broad and strong absorption peak at 3363 cm^−1^ corresponds to the stretching vibration of amino (−NH_2_) or acylamino groups. The peaks at 2947 cm^−1^ and 2883 cm^−1^ are attributed to the antisymmetric vibration of carboxylate/sulfonate groups and the aliphatic C−H stretching vibration, respectively. Characteristic absorptions of amide groups appear at 1667 cm^−1^ and 1546 cm^−1^, corresponding to C=O stretching and N−H bending vibrations, respectively. Moreover, the strong peak at 1311 cm^−1^ is assigned to the deformation vibration of methylene/methyl groups, while absorptions at 1129 cm^−1^ and 1021 cm^−1^ indicate the presence of C=O and S=O bonds, respectively. In the low-frequency region, the doublet splitting at 811 cm^−1^ and 761 cm^−1^ is a characteristic signal of the in-plane bending vibration of S–O–H in sulfonic acid groups (−SO_3_H). These results demonstrate that the phenolic crosslinker and polymer molecules interact synergistically through hydrogen-bond association and covalent crosslinking, forming a stable three-dimensional network structure. This dual-crosslinking mechanism of “rigid covalent bonds–dynamic hydrogen bonds” is key to the gel’s combined high modulus and good toughness at elevated temperatures, confirming the successful synthesis of a high-temperature-resistant resin–gel.

### 2.2. Evaluation of the Applicability of High-Temperature-Resistant Resin–Gel Plugging System

#### 2.2.1. Contamination Resistance of Resin–Gel Plugging System

**Figure 7:**
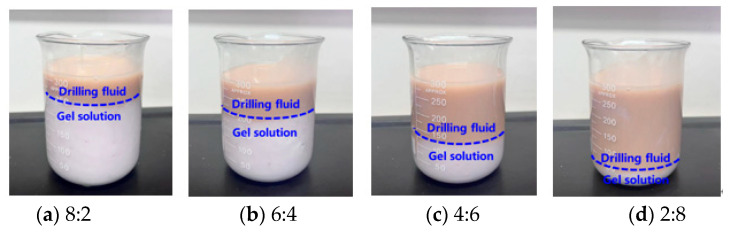
Static mixing of drilling fluid with resin–gel solution at different volume ratios.

The experimental results show that the resin–gel solution (density 1.10 g·cm^−3^) and 4.0% bentonite-based slurry (density 1.05 g·cm^−3^) show a clear layered interface after mixing (Figure 7), indicating that the two are well-matched and the resin–gel solution has excellent “water repellency” properties. At 150 °C, the apparent viscosity of the drilling fluid-contaminated gel system only decreased from 7928 mPa·s to 7162 mPa·s (a reduction rate of 9.7%), while retaining over 87% of its initial storage modulus.

**Figure 8:**
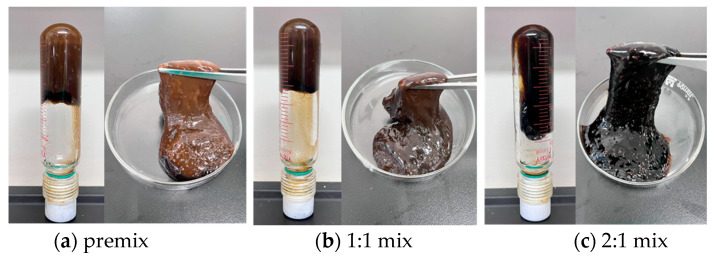
Gel state before and after mixing the resin–gel solution with the base paste.

**Figure 9:**
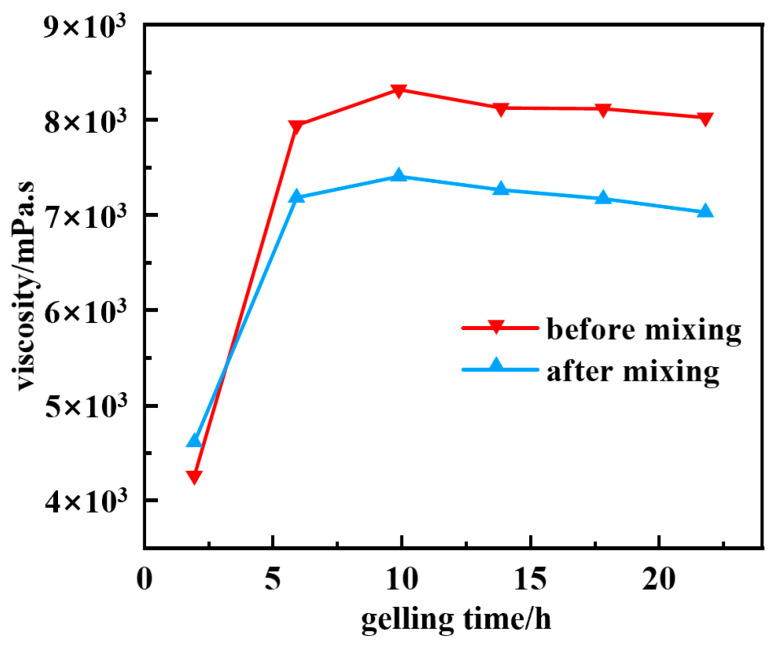
Viscosity change before and after mixing of 150 °C resin–gel solution and base paste.

At the same time, the system with different concentration gradient compounding was put into high-temperature 150 °C conditions for aging and gel formation, and the anti-drilling fluid contamination performance of the resin–gel was evaluated by testing the viscosity of the system and the strength of the gel formation after compounding. The experimental results are shown in Figure 8 and Figure 9. Owing to immiscibility, a 1:1 mixture of resin–gel solution and drilling fluid showed an apparent viscosity drop from 7928 to 7162 mPa·s, with minimal impact on gel strength. This confirms excellent anti-contamination ability. The mechanism analysis reveals that the drilling fluid particles in the resin–gel solution of mixed drilling fluid lead to a certain degree of compression within the system, making the molecular chains entangled with each other. Moreover, the thermal buffer effect produced by the particle accumulation layer in the drilling fluid enhances the thermal degradation activation energy of the gel, which is manifested by a slower tendency to reduce its viscosity during high-temperature aging.

#### 2.2.2. Weighting Properties of Resin–Gel Plugging System

**Figure 10:**
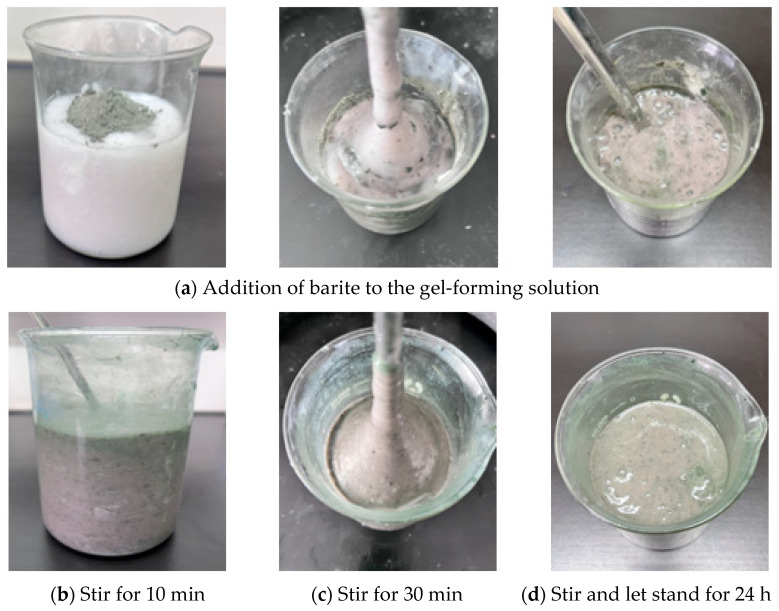
Resin–gel solution weighting process.

In order to ensure construction safety and enhance the sealing effect during the deep wellbore sealing operation, this study conducted density optimization experiments on the preferred resin–gel plugging system. The resin–gel solution was prepared according to the optimized formulation, and the density of the gel solution was increased to 1.3 g·cm^−3^, 1.5 g·cm^−3^, and 2.0 g·cm^−3^ by using weighted materials (barite, magnetite powder, and calcium carbonate), respectively. The weighting process is illustrated in Figure 10, and the barite could be fully mixed into the solution under continuous stirring. The static stability assessment showed that the mixed solution did not show obvious phase separation phenomenon after 24 h of standing at 25 °C, and the barite particles were still uniformly distributed in the solution. The gel formation test at 150 °C showed that the weighting process not only solved the construction problems but also significantly improved the sealing performance of the resin–gel, and its enhanced density not only ensured the safety of the construction but also optimized the gel formation time and the temperature resistance by changing the chemical environment of the system, which is suitable for the sealing operation of high-pressure gas wells. Enhanced density ensures construction safety and optimizes gelation time and temperature resistance by altering the system’s chemical environment. This offers a reliable technical solution for sealing high-pressure gas wells.

**Figure 11:**
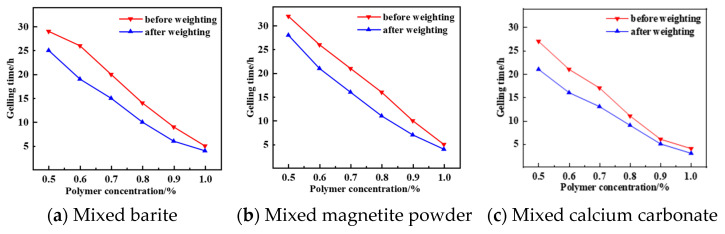
Comparison of gel formation time before and after resin–gel solution weighting.

**Figure 12:**
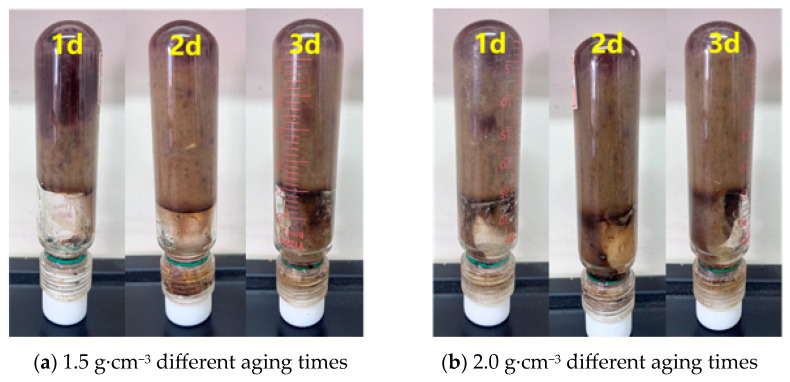
Diagram of the aging state of the barite-weighted resin–gel.

**Figure 13:**
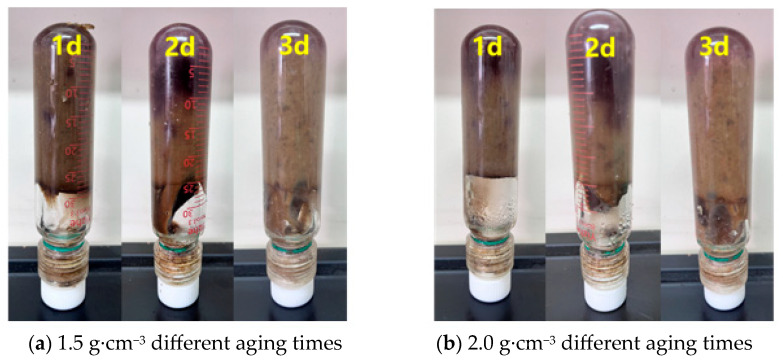
Diagram of the aging state of the magnetite-weighted resin–gel.

**Figure 14:**
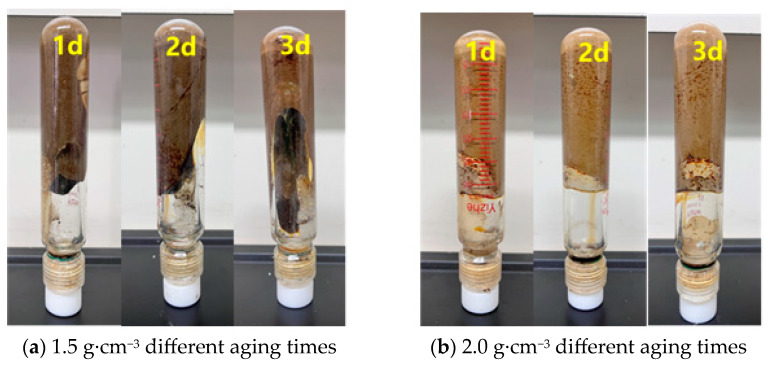
Diagram of the aging state of the calcium carbonate-weighted resin–gel.

As can be seen from Figure 11, Figure 12, Figure 13 and Figure 14, the gel formation time of the gel system after weighting with barite, magnetite, and calcium carbonate decreases with the increase in polymer concentration. And with the increase in polymer concentration, the difference between the gel formation time before and after weighting gets smaller and smaller: the difference is 3 h when the polymer concentration is 0.5%, and the difference is reduced to 1 h when the concentration is 1.0%. The weighting materials act primarily as physical fillers and partial physical crosslinking points. Their presence introduces steric hindrance, which delays gelation by reducing the probability of polymer–crosslinker encounters. Higher polymer concentrations mitigate this delay by increasing chain entanglement and enhancing network formation kinetics. Meanwhile, the type and loading of weighting materials, together with polymer concentration, jointly determine the gel’s microstructure—affecting both its formation kinetics and its final mechanical integrity.

**Figure 15:**
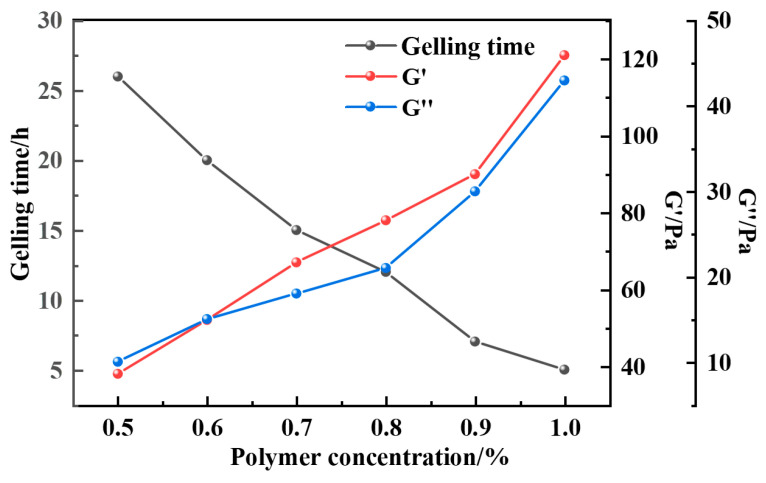
Gel-forming properties of the resin–gel weighted with barite to 1.5 g·cm^−3^.

**Figure 16:**
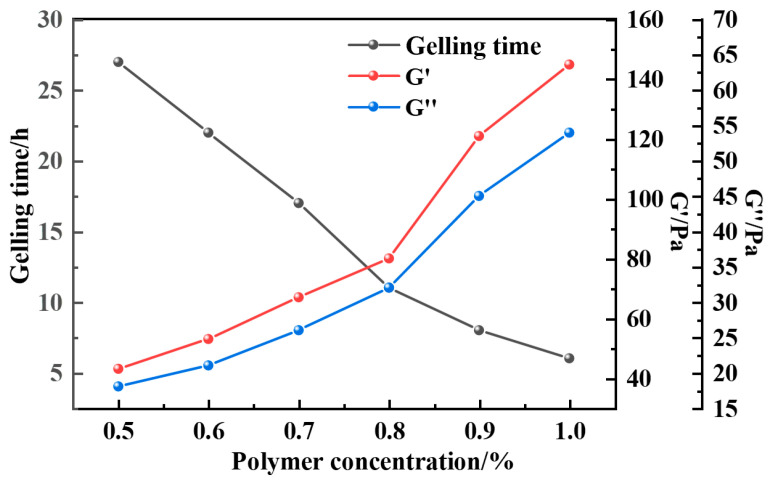
Gel-forming properties of the resin–gel weighted with magnetite to 1.5 g·cm^−3^.

**Figure 17:**
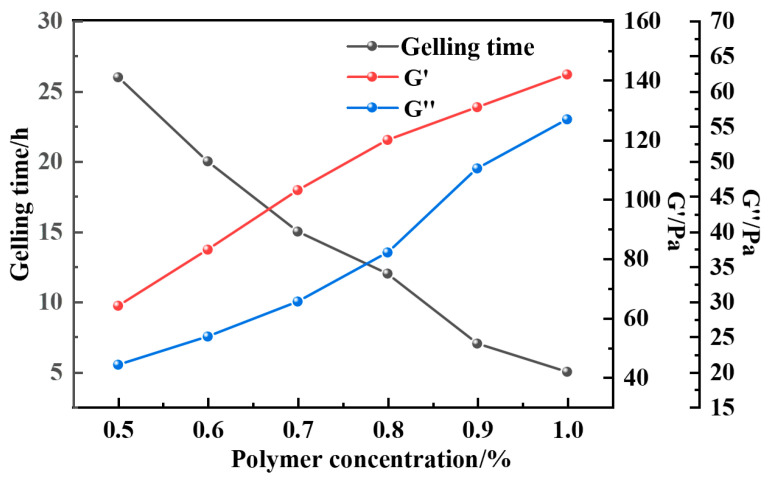
Gel-forming properties of the resin–gel weighted with calcium carbonate to 1.5 g·cm^−3^.

As can be seen in Figure 15, Figure 16 and Figure 17, the gel formation time is relatively long when the polymer concentration is 0.5%. As the polymer concentration increases, the distance between the polymer molecules decreases and the intermolecular interactions increase. The polymer molecules are able to form a three-dimensional network structure faster, which results in a shorter gelation time. It can be observed from the figure that the gel formation time decreases significantly when the polymer concentration increases from 0.5% to 1.0%. The viscosity and strength of the system were significantly increased after aging the gel at the high temperature of 150 °C with a barite-aggravated gel density of 1.5 g·cm^−3^. The G′ and G″ of the gel system with a polymer concentration of 1.0% were 122 Pa and 44 Pa, respectively.

The density of the gel system was increased to 1.5 g·cm^−3^ and 2.0 g·cm^−3^ using magnetite, and the strength of the gel system after aging at the high temperature of 150 °C was also greatly improved, and the energy storage modulus and loss modulus of the gel with a density of 1.5 g·cm^−3^ were 148 Pa and 55 Pa, and the strength of the system was up to H class.

The gel-forming strength of the gel system was also significantly improved after weighting with calcium carbonate. Because of the lower density of calcium carbonate compared with barite and magnetite powder, the energy storage modulus and loss modulus when the density of the gel system was weighted to 1.3 g·cm^−3^ were 144 Pa and 56 Pa, respectively, and the strength of the system was still up to H class.

It can be seen through the use of barite-weighted gel after the change in the complex modulus of the gel system that the system selects barite as the weighted material to join and its viscosity is improved, but the elasticity of the colloid compared with the weighted gel undergoes a substantial decrease. This situation indicates that barite is added to the system in the form of physical mosaic. The systems after weighting to different densities are able to form stronger gels after a longer aging time, and all of them show good stability performance. This performance advantage makes them able to meet the engineering demand for long-lasting containment of high-density drilling fluids in deep wells and significantly improve the success rate of containment. The study further reveals that weighting materials such as barite are not entirely inert within the system. Physical adsorption between the particle surfaces and polymer chains can create additional physical crosslinking points, which contributes to enhancing the modulus and suppressing syneresis. However, the steric hindrance introduced by excessive particle loading can also delay gelation. This indicates that the interfacial interactions and dispersion state of the materials are critical factors for performance optimization.

#### 2.2.3. High-Temperature Stability of Resin–Gel Plugging System

In this study, the dehydration performance of the preferred resin–gel plugging system (0.5% NEP + 0.5% DEP + 0.6% HMTA + 0.3% catechol + 25% urea–formaldehyde resin) was systematically evaluated for the high-temperature and high-pressure wellbore sealing needs. The experimental data showed that, under the condition of 2 MPa differential pressure, the EHV water loss of the unweighted resin–gel solution after aging for 0.5 d at the high temperatures of 130 °C and 150 °C was 6.1 mL·100 mL^−1^ and 7.3 mL·100 mL^−1^, respectively; the EHV water loss after aging for 3 d was 7.2 mL·100 mL^−1^ and 8.2 mL·100 mL^−1^, respectively; and the EHV water loss after aging for 9 d was 8.6 mL·100 mL^−1^ and 9.0 mL·100 mL^−1^, respectively. The EHV water loss after aging for 0.5 d at 130 °C and 150 °C with a weighting of 1.5 g·cm^−3^ was 5.3 mL·100 mL^−1^ and 6.3 mL·100 mL^−1^, respectively; the EHV water loss after aging for 0.5 d was 5.3 mL·100 mL^−1^ and 6.3 mL·100 mL^−1^, respectively; after aging for 3 d, the EHV water loss was 6.6 mL·100 mL^−1^ and 7.6 mL·100 mL^−1^, respectively; and after aging for 9 d, the EHV water loss was 7.8 mL·100 mL^−1^ and 8.4 mL·100 mL^−1^, respectively.

**Figure 18:**
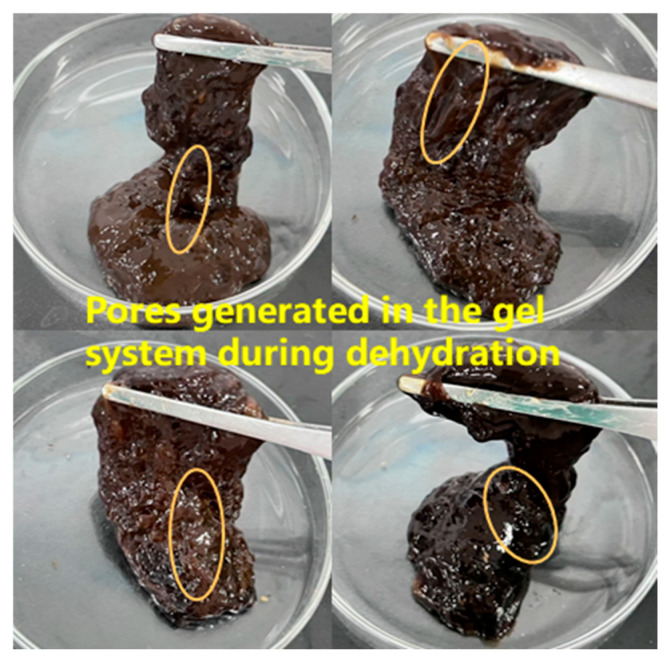
State of the resin–gel after water loss under high temperature and pressure.

According to the experimental data, it can be seen that both temperature and density have a more obvious effect on the dehydration rate of the system. The amount of gel dehydration increases with the increase in temperature, which is due to the hydrolysis of polymer macromolecule chains in the gel at high temperatures, resulting in the dehydration and contraction of the gel. Under the condition of the high temperature of 140 °C, when the gel density increased to 1.5 g·cm^−3^, the amount of dehydration decreased from 6.5 mL·100 mL^−1^ before the original exacerbation to 6.1 mL·100 mL^−1^, and the amount of gel dehydration decreased with the increase in the gel density. The gel state after high-temperature and high-pressure dehydration is shown in Figure 18. This situation occurs because the barite particles can be used as physical crosslinking points to increase the cohesion of the gel so that it is less likely to be destroyed under high temperature and high pressure, which in turn reduces the amount of water loss, and the barite particles can fill in the pore structure of the gel to reduce the pore space inside the gel. Under high temperature and high pressure, the free water in the gel will try to seep out through the pores. The filling effect of barite can hinder the path of water exudation, thus reducing the amount of water loss.

By analyzing the data in Table 3 and comparing the high-temperature dehydration volume with the high-temperature and high-pressure dehydration volume at the same temperatures, it is found that pressure has a relatively minor effect on the dehydration behavior of the gel system. Temperature is the dominant factor influencing gel dehydration. Under the condition of 130 °C, the total high-temperature and high-pressure dehydration volume of the unweighted gel after aging for 3 days is 7.2 mL·100 mL^−1^, while at 150 °C, it increases slightly to 8.2 mL·100 mL^−1^. The values for the weighted gel are even lower, indicating enhanced stability. Therefore, this gel system is suitable for borehole plugging applications in open-hole intervals.

#### 2.2.4. Gel-Breaking Performance of Resin–Gel Plugging System

The gel system developed in this study shows excellent high-temperature gel-breaking performance, which effectively solves the technical problem of traditional gels that are difficult to break under high-temperature conditions through the oxidative degradation mechanism. As shown in Table 4, when 15% ammonium persulfate is used as the gel-breaking agent, a gel-breaking rate of more than 70% can be realized in only 12 h at a high temperature of 150 °C, and the gel-breaking rate can reach 99.8% in 24 h. During the gel-breaking process, the gel mass drops sharply from 30 g to 2.1 g. This rapid, controllable gel-breaking arises from ammonium persulfate’s oxidative attack on amide groups and the C–C backbone, involving polymer backbone cleavage, crosslink destruction, and hydrolysis promotion. This gel-breaking technology has the advantages of easy operation (only immersion is required), low cost (conventional oxidizing agent is used) and high efficiency, which is especially suitable for the rapid unsealing of high-temperature and high-pressure wellbores after operation and provides a reliable temporary sealing solution for the development of oil and gas fields.

**Figure 19:**
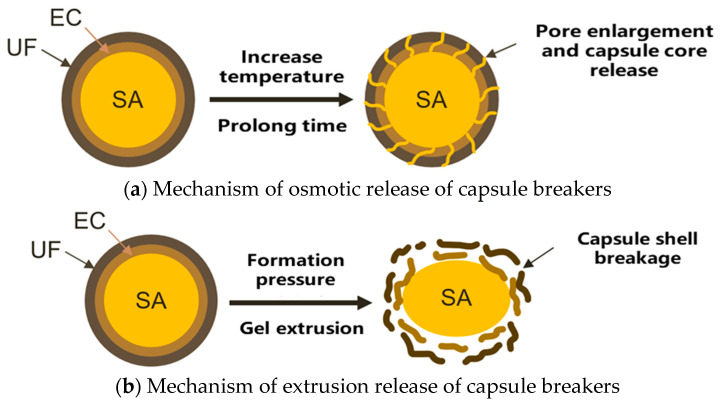
Mechanism of release of capsule breakers.

In this study, high-temperature-resistant capsule gel-breaking agents with a bilayer shell structure were successfully prepared by an innovative composite process. The specific preparation process is as follows: Firstly, the primary capsules of ethyl cellulose/sulfamic acid (EC/SA) were prepared by an oil phase separation method with a core-to-wall mass ratio of 10:3, and a high encapsulation rate of 85.3% was achieved. Then, the in situ polymerization method was adopted, and the hot-melt resin condensed on the surface of EC under the action of Tween-80 emulsifier (mass ratio of 1.5:1) to form the double-layer capsule structure of “ethyl cellulose–hot-melt resin”. Characterization results show that the resulting capsule has the following excellent properties: (1) regular shape, with a sphericity of more than 92%; (2) dense capsule structure, with no visible defects; (3) outstanding temperature resistance, which can withstand high temperatures up to 150 °C; (4) controllable slow-release performance, which can be achieved by adjusting the thickness of the bilayer capsule (20–50 μm) and the ratio of the composition to achieve the precise release of the capsule for 12–72 h. This product successfully solves the technical problem of premature release of traditional gel-breaking agents under high-temperature conditions and provides a reliable temporary plugging solution for the transformation of high-temperature reservoirs in deep wells. The microcapsule gel breaker can be directly mixed into the gel system, and the slow release of the gel breaker can be realized through temperature control after the gel is formed (Figure 19). When the ambient temperature exceeds the glass transition temperature (Tg), the thermoplastic resin shell softens, enhancing polymer chain mobility and creating diffusion channels. This thermal activation initiates two subsequent release pathways: (a) osmotic release, driven by concentration gradients across the softened shell, and (b) extrusion release, resulting from mechanical stress or internal pressure buildup. These mechanisms operate synergistically to achieve controlled and efficient release of the gel-breaking agent under high-temperature conditions. The technical advantages are mainly reflected in the following: (1) the release kinetics can be precisely regulated: by adjusting the parameters such as capsule thickness (50–200 μm), shell material composition (e.g., polyethylene/paraffin ratio), and medium temperature (90–150 °C), an adjustable release window of 12–72 h can be realized; (2) environmentally responsive release: when the temperature of the system exceeds the melting point of the capsule (usually set as the reservoir temperature), the capsule melts and releases the sulfamic acid; (3) high gel-breaking efficiency: the released sulfamic acid breaks the ether and amide bonds in the polymer molecule through protonation. Experiments have confirmed that this technology can achieve over a 95% gel breakage rate within a set time, while avoiding the problem of premature failure of conventional gel breakers, which provides a new technical means for intelligent completion and temporary plugging operations.

This study systematically investigated the gel-breaking kinetics of a 10% capsule-based gel breaker under different temperature conditions. Experimental results indicate that under high-temperature conditions (150 °C), the capsule breaker exhibits well-defined delayed-release behavior. As shown in Table 5, the capsules maintained structural integrity during the first 15 days (release rate < 5%), with the elastic modulus remaining stable at approximately 103 Pa. Upon triggering gel-breaking on the 15th day, the elastic modulus rapidly dropped to 50 Pa within 48 h (a decrease of 51.5%), and by the 21st day, the release rate of the breaker exceeded 90%. This temperature-responsive release mechanism is attributed to the phase transition behavior of the capsule shell material: when the ambient temperature reaches the critical point, the thermoplastic resin shell softens (Tg ≈ 145 °C), enhancing the mobility of polymer chain segments and forming diffusion channels that enable the release of sulfamic acid. Notably, by adjusting the coating thickness (20–50 μm) and crosslinking density, the gel-breaking time can be precisely controlled within a 7–21 day window. This technique provides a programmable gel-breaking solution for high-temperature reservoirs, effectively addressing the challenge of uncontrolled gel-breaking timing associated with conventional breakers.

**Figure 20:**
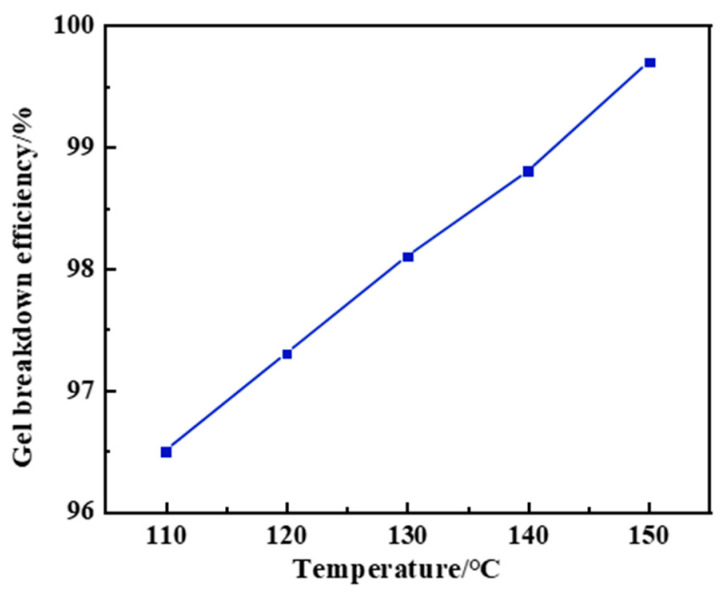
Gel-breaking efficiency of capsule breakers at different temperatures.

**Figure 21:**
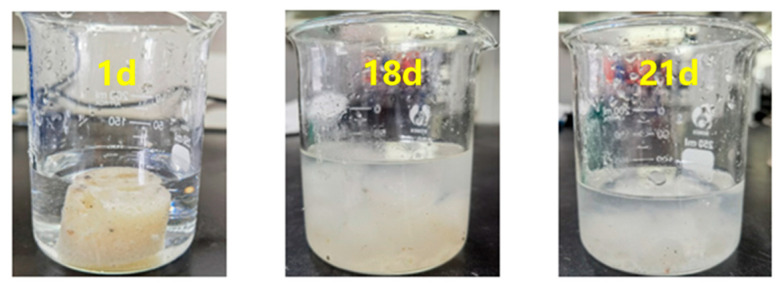
Gel-breaking effect of capsule breakers with different aging time at 150 °C temperature.

**Figure 22:**
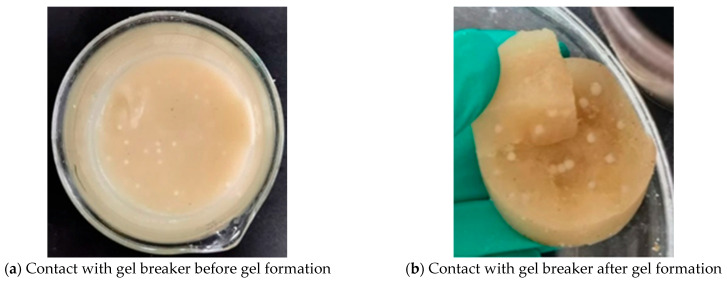
Effect of contact between capsule breaker and resin–gel before and after gel formation.

This study systematically evaluated the gel-breaking performance of a 10% capsule gel breaker under different temperature conditions. Experimental results showed that under high-temperature conditions of 150 °C, the breaker exhibited excellent sustained-release properties and gel-breaking efficiency: after 21 days of action, the gel-breaking rate of the system reached 99.7% (Figure 20). As shown in Figure 21 and Figure 22, interfacial contact experiments confirmed that direct interaction between the capsule gel breaker and the gel system significantly accelerated the degradation rate. The mechanism primarily involves: (1) temperature-triggered softening of the capsule shell (Tg ≈ 145 °C), enabling controlled release of the gel breaker, (2) the released sulfamic acid breaking the polymer chains via protonation, (3) redox reactions that destroy the crosslinked network structure. A key advantage of this technology lies in its ability to enable integrated “injection–plugging–gel-breaking” operations. The release kinetics of the microcapsules, adjustable through shell thickness and crosslinking density, are effectively synchronized with the high-temperature aging process of the gel network, enabling precise programming of the gel-breaking time within a 7- to 21-day window. This approach successfully resolves the core technical challenge associated with conventional oxidizing gel breakers, which often suffer from uncontrollable activation timing and premature degradation at high temperatures. The capsule-based gel breaker can be co-injected with the gel system into the wellbore, where downhole temperature and pressure conditions automatically trigger the gel-breaking process, providing an efficient and reliable temporary plugging-removal solution for high-temperature reservoir operations.

#### 2.2.5. Plugging Performance of Resin–Gel Plugging System

**Figure 23:**
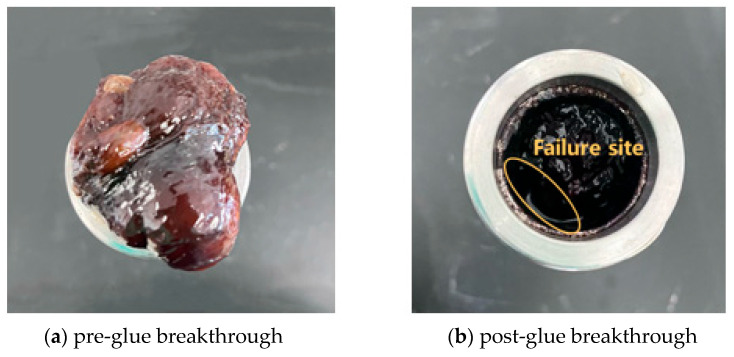
Before and after gel segment plug breakthrough.

This study systematically evaluated the pressure-bearing plugging performance of the resin–gel using a high-temperature and high-pressure plugging–displacement apparatus. A sand-packed tube model with an inner diameter of 38 mm and a length of 50 cm was used to test the plugging performance of the gel system after aging for 1 to 3 days at 130 °C and 150 °C. As shown in Table 6, under water-flooding conditions, the plugging capacity after aging for 1 and 3 days at 130 °C was 0.178 MPa·m^−1^ and 0.148 MPa·m^−1^, respectively, while at 150 °C, the values decreased to 0.156 MPa·m^−1^ and 0.108 MPa·m^−1^. Under gas-flooding conditions, the plugging capacity after aging for 1 and 3 days at 130 °C was 0.168 MPa·m^−1^ and 0.100 MPa·m^−1^, respectively, and further declined to 0.158 MPa·m^−1^ and 0.066 MPa·m^−1^ at 150 °C. These results confirm a consistent decrease in the plugging performance of the gel system with increasing temperature and aging time. Among these factors, temperature has a more pronounced weakening effect on the plugging ability, especially under gas-flooding conditions. This provides important guidance for optimizing parameters in plugging operations for high-temperature reservoirs. Additionally, as illustrated in Figure 23, the failure site after gel breakthrough primarily exhibits cohesive failure, characterized by internal fracture of the gel network rather than detachment from the sand-pack tube wall. This indicates that under conditions of 150 °C and high pressure, the internal crosslinking strength of the gel becomes the limiting factor, rather than its adhesion to the formation. Further enhancement of the gel’s crosslinking density or the incorporation of reinforcing agents could improve its pressure-bearing capacity in ultra-high-temperature applications.

Table 7 further compares the performance of the developed plugging agent with recently reported systems in the literature. The comparison results demonstrate that the resin–gel plugging agent developed in this study maintains a high storage modulus and structural integrity at 150 °C, exhibiting superior temperature resistance compared to most reported gel systems. Furthermore, owing to the microcapsule-controlled gel-breaking technology, the system achieves a gel-breaking rate exceeding 99.7% within 21 days, demonstrating enhanced controllability of degradation and better adaptability to reservoir conditions.

## 3. Conclusions

This study addresses the challenge of high-temperature gas channeling in karst-fractured reservoirs by successfully developing and systematically evaluating a high-temperature-resistant, high-strength, and controllably degradable resin–gel plugging system. The main conclusions are as follows:

(1) A dual-crosslinked network was constructed based on an AMPS/NVP copolymer matrix (molar ratio 3:1) and phenolic resin, while polyamide microcapsules (shell thickness 1.2–1.8 μm) were incorporated to achieve controlled release of the gel breaker. The optimized formulation consists of 0.5% NEP + 0.5% DEP + 0.6% HMTA + 0.3% catechol + 25% resin curing agent. After aging at 150 °C for 14 days, the system retains more than 90% of its storage modulus and achieves a gel-breaking rate of 99.7% within 21 days, overcoming the technical limitations of conventional gels regarding insufficient temperature resistance and difficulty in gel-breaking.
(2) Under 150 °C conditions, the gel exhibits a G′ ≥ 125 Pa and a compressive strength > 18 MPa (at 15% strain). High-temperature and high-pressure tests (2 MPa differential pressure) show a dehydration rate below 5% (e.g., the weighted system at 1.5 g·cm^−3^ exhibits a dehydration of only 8.4 mL·100 mL^−1^ after aging at 150 °C for 9 days). Moreover, the system demonstrates excellent contamination resistance: after mixing with drilling fluid, the viscosity decreases by less than 9.7%, while the retention rate of the storage modulus exceeds 87%.
(3) Under high-pressure conditions at 150 °C, the plugging efficiency of the system exceeds 92% for fractures with widths ranging from 0.1 to 2 mm. After 3 days of aging under gas-drive conditions, the equivalent pressure-bearing capacity per 100 m remains as high as 17 MPa. Compared with similar imported systems, the cost is reduced by approximately 41%. This system can effectively mitigate high-temperature gas channeling in injection wells of fracture-cavity reservoirs and holds promising potential for extension to applications such as temporary plugging in shale gas fracturing and sealing in geothermal reservoirs.

## 4. Materials and Methods

### 4.1. Experimental Materials

The polymers NEP and DEP, with molecular weights in the range of 3.0 × 10^6^–5.0 × 10^6^ Da, were supplied by Shandong Nuor Chemical Co., Ltd., Jining, China. Hexamethylenetetramine (HMTA; AR grade; ≥99%) was obtained from Shanghai Macklin Biochemical Technology Co., Ltd., Shanghai, China. Catechol (AR grade, ≥99%) was purchased from Shanghai Aladdin Biochemical Technology Co., Ltd., Shanghai, China. The urea–formaldehyde resin curing agent was provided by Zibo Ocean Industry Co., Ltd., Zibo, China. Ammonium persulfate (AR grade, ≥98%) and hydrochloric acid (CP grade, 36.0–38.0%) were acquired from Sinopharm Chemical Reagent Co., Ltd., Shanghai, China. All aqueous solutions and solvents used in the experiments were prepared using laboratory-produced deionized water with a resistivity of 18.25 MΩ·cm.

### 4.2. Construction of High-Temperature-Resistant Resin–Gel Plugging System

The polymers were dissolved in deionized water to formulate a masterbatch solution at a concentration of 1.0% (*w*/*v*; all concentrations in the text are by mass volume). Mechanical stirring was used to mix the polymer solution thoroughly to ensure complete dissolution, followed by static aging at room temperature (25 ± 1 °C) for 24 h to ensure adequate hydration of the polymer and relaxation of the molecular chains. Afterwards, the master polymer solution was diluted with deionized water to obtain the test solution at the desired experimental concentration. During the dilution process, low-speed stirring was maintained to avoid mechanical degradation of the polymer chains.

### 4.3. Characterization of Physicochemical Properties of High-Temperature-Resistant Resin–Gel Plugging System

#### 4.3.1. Infrared Spectral Characterization of Resin–Gel Plugging System

The dried gel particles were mixed with potassium bromide (KBr) at a mass ratio of 1:99, and after thorough grinding, the transmission infrared test tablets were pressed under high pressure using a tablet press. A Fourier transform infrared (FTIR) spectrometer (IRTRacer-100, SHIMADZU, Kyoto, Japan) was used to scan in the wavelength range of 400–4000 cm^−1^, 35 scans were accumulated to improve the spectral signal-to-noise ratio, and the vibrational information of the characteristic functional groups of gel molecules was finally obtained, which was used to analyze their chemical structure and intermolecular interactions.

#### 4.3.2. Thermogravimetric Analysis of Resin–Gel Plugging System

The thermal stability of the gel samples was tested using the TGA 2 SF thermogravimetric analyzer (METTLER TOLEDO, Zurich, Switzerland) under the conditions of 300 Pa test pressure and 20 cm^3^/min inert gas flow rate at a constant heating rate of 10 °C/min. After the instrument was preheated and zeroed before the experiment, an appropriate amount of dried sample was placed in a crucible, and the dynamic thermal analysis was performed in the temperature range of 30–600 °C. The thermogravimetric (TG) curves were obtained by continuously monitoring the changes in the sample mass to determine the thermal stability and decomposition characteristics of the gel.

#### 4.3.3. Evaluation of Gel Formation Time and Gelling Effect of Resin–Gel Plugging System

**Figure 24:**
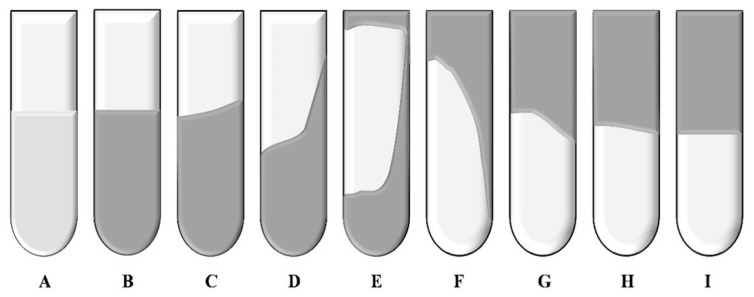
Schematic diagram of different gel strength codes [36].

The sealed ampoule was placed in a constant-temperature blast drying oven for the crosslinking reaction, and the Sydansk bottle test method was used to observe the change in the gel flow state with time to determine the gel formation characteristics. The initial gel formation time was recorded when the gel strength reached grade D for the first time (a small amount of gel could not flow when the ampoule was turned over), and the time when the gel strength reached a stable state at high temperature was recorded as the final gel formation time (GT). Gel strength was classified into 9 grades (A to I) based on flow behavior: grade A represents the initial state with no viscosity change; grades B and C correspond to high fluidity and partial fluidity, respectively; grades D, E, and F show a gradual decrease in fluidity; grades G and H show that the gels can only flow partially or deform slightly; and grade I is a completely rigid and non-fluid state, which is specifically characterized by the lack of flow and the unchanged surface morphology of the gels when the ampoule bottle is turned over. This grading system (Figure 24) objectively characterizes the transition of the gel from liquid to solid by quantifying the degree of flow and deformation.

#### 4.3.4. Rheo-Mechanical Testing of Resin–Gel Plugging System

A HAAKE Mars60 rheometer (Thermo Fisher Scientific, Wilmington, DE, USA) combined with a P35 rotor (plate gap 0.052 mm) was used for the rheological analysis of polymer gels under different gel-forming conditions, and the energy storage modulus (G′) and loss modulus (G″) were measured by the dynamic oscillatory mode (fixed at 1 Hz, with a stress scan of 0.1–100 Pa), while the shear mode (rate 0.1–100 s^−1^) was used for measurement. The steady state rheological behavior of the gels was tested to evaluate their viscoelasticity, network structural strength and shear thinning properties.

### 4.4. Evaluation of the Applicability of High-Temperature-Resistant Resin–Gel Plugging System

#### 4.4.1. Evaluation of Anti-Pollution Performance of Resin–Gel Plugging System

During the field application of the polymer gel system, the effect of formation water mineralization (Na^+^ and Ca^2+^ concentration) on the gel-forming properties (gel-forming time and gel-forming strength) needs to be investigated in order to assess the inhibition or enhancement of the gel network structure by the high-salt environment. Meanwhile, since the gel solution needs to be mixed with drilling fluid for injection, it is necessary to test the compatibility of the two and evaluate the effect of drilling fluid on the gel system through rheological tests (e.g., storage modulus G′ analysis) and gel formation time monitoring after mixing at different volume ratios, so as to optimize the construction plan and ensure that the gel can maintain the required plugging or drive modulation ability in the complex downhole environment.

#### 4.4.2. Evaluation of Solution Weighting Properties of Resin–Gel Plugging System

By adding temperature-resistant polymer and weighting material (barite powder, particle size 0.150–0.180 mm) synchronously, the gel density is increased to 1.5–2.0 g·cm^−3^ in stages by the process of rapid feeding and high-speed mixing; priority was given to barite, and its weighting performance is evaluated by comparing the change in gel formation time of the system before and after weighting. If the extension of gel formation time is controllable (≤30%), the gel strength is maintained at ≥2.5 MPa and the temperature resistance is ≥120 °C after the density is increased, then it indicates that the weighting agent is uniformly dispersed and does not destroy the chemical stability of the system.

#### 4.4.3. Evaluation of High-Temperature Stability of Resin–Gel Plugging System

In order to evaluate the high-temperature stability of the polymer gel system, the gel dehydration rate can be used for semi-quantitative analysis. The specific method is as follows: place the ampoule that has been formed into a gel in a constant-temperature blast oven at 120–160 °C, take it out every 3 days and weigh its mass, and calculate the dehydration rate S, i.e., the ratio of the amount of dehydrated water to the mass of the initial gel, by recording the change in the mass of the gel after it has been dehydrated from water. This method can effectively reflect the dehydration and shrinkage trend of the gel in a high-temperature environment, and then evaluate its structural stability.

#### 4.4.4. Evaluation of Sealing and Breaking Performance of Resin–Gel Plugging System

**Figure 25:**
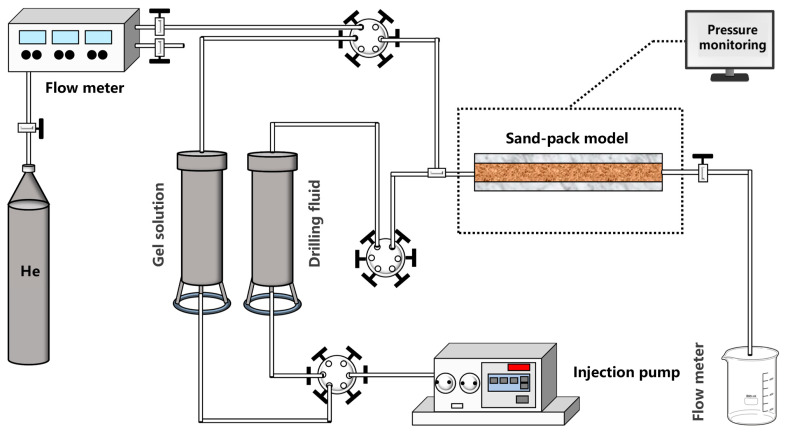
Schematic diagram of the experimental setup for the polymer gel plugging experiment.

A high-temperature and high-pressure plugging test setup was used to test the effectiveness of the blocking of the gel plugging agent, and Figure 25 depicts the experimental setup used to evaluate the blocking performance of the highly stable polymer gel under high-temperature conditions. The sand-filled pipe (inner diameter of 38 mm, length of 50 cm, and inlet and outlet diameter of 6 mm) was used as a simulated leakage channel model, and slurry water was pumped in at a slurry injection rate of 5 mL/min under constant temperature conditions. The breakthrough pressure of the gel plugging layer under different aging times was continuously monitored to quantitatively characterize the plugging strength of the polymer gel system and its high-temperature stability, and the value of the breakthrough pressure directly reflects the mechanical properties and scour resistance of the gel network structure.

## Figures and Tables

**Table 1:** Viscosity temperature data at NEP:DEP = 1:1.

Concentration (%)	Viscosity (mPa·s)					
	80 °C	100 °C	120 °C	140 °C	160 °C	180 °C
0.6	962	741	623	521	438	316
0.8	1204	1142	942	812	743	621
1.0	1763	1309	1183	1063	951	903
1.2	1941	1567	1344	1251	1182	1094

**Table 2:** Effect of polymer compounding on resin–gel strength.

Polymer Type	Compounding Ratio	Phenol Concentration (%)	Aldehyde Concentration (%)	Gelation Time (h)	Gel Strength
NEP/DEP	1:2	0.2	0.4	15	D
	2:1	0.2	0.4	12	F
	1:1	0.2	0.4	8	H

**Table 3:** Water loss of resin–gel system at high temperature and pressure.

Serial Number	Density (g·cm^−3^)	Temperature (°C)	Aging Time (d)	High-Temperature Dehydration (mL·100 mL^−1^)	High-Temperature and High -Pressure Dewatering (mL·100 mL^−1^)
1	1.1	120	0.5	5.8	6.1
2	1.1	120	3	6.7	7.2
3	1.1	120	9	7.9	8.6
4	1.5	120	0.5	5.1	5.3
5	1.5	120	3	6.2	6.6
6	1.5	120	9	7.0	7.8
7	1.1	140	0.5	6.5	7.3
8	1.1	140	3	7.6	8.2
9	1.1	140	9	8.3	9.0
10	1.5	140	0.5	6.1	6.3
11	1.5	140	3	7.0	7.6
12	1.5	140	9	7.9	8.4

**Table 4:** Resin–gel variation with time for room temperature and 120 °C immersion of 15% ammonium persulfate.

Temperature (°C)	Weight (g)					
	0 h	6 h	12 h	18 h	24 h	48 h
25	30	16.5	9.8	3.1	1.3	0.3
120 °C	30	12.5	4.7	2.3	0.8	0.2

**Table 5:** Modulus of elasticity of gel after breaking of capsule breakers at different temperatures.

System	Modulus (Pa)					
	25 °C	110 °C	120 °C	130 °C	140 °C	150 °C
Added glue breakers	125	102	95	84	70	50
No glue breakers	125	121	118	113	108	103

**Table 6:** Sealing strength of resin–gel.

Serial Number	Gel Segment Plug Length (cm)	Temperature (°C)	Aging Time (d)	Pressure-Bearing Capacity (MPa)	Pressure-Bearing Per 100 m (MPa)
1 (water-driven)	50	130	1.0	0.089	17.8
2 (water-driven)	50	130	2.0	0.083	16.6
3 (water-driven)	50	130	3.0	0.074	14.8
4 (water-driven)	50	150	1.0	0.078	15.6
5 (water-driven)	50	150	2.0	0.071	14.2
6 (water-driven)	50	150	3.0	0.054	10.8
7 (gas-driven)	50	130	1.0	0.084	16.8
8 (gas-driven)	50	130	2.0	0.061	12.2
9 (gas-driven)	50	130	3.0	0.050	10.0
10 (gas-driven)	50	150	1.0	0.079	15.8
11 (gas-driven)	50	150	2.0	0.053	10.6
12 (gas-driven)	50	150	1.0	0.042	8.4

**Table 7:** Performance comparison of plugging agents.

No.	Plugging Agent	Temperature Resistance (°C)	Gel Breaker	Gel-Breaking Rate (%)	Ref.
1	Resin–Gel	150	microcapsule breaker	99.7	This study
2	PVA/XC Gel	110	potassium persulfate	91	Liu et al. [33]
3	PAM Gel	140	ammonium persulfate	95	Li et al. [34]
4	CNF/PAM nanocomposite gel	140	ammonium persulfate	91.8	Yang et al. [35]

## Data Availability

Data is contained within the article.

## References

[1] Hu Y., Hao M., Chen G., Sun R., Li S. (2019). Technologies and practice of CO_2_ flooding and sequestration in China. Pet. Explor. Dev..

[2] Thakkar A., Raval A., Chandra S., Shah M., Sircar A. (2020). A comprehensive review of the application of nano-silica in oil well cementing. Petroleum.

[3] Zhao G., You Q., Tao J., Gu C., Aziz H., Ma L., Dai C. (2018). Preparation and application of a novel phenolic resin dispersed particle gel for in-depth profile control in low permeability reservoirs. J. Pet. Sci. Eng..

[4] Qing Y., Yefei W., Wei Z., Ziyuan Q., Fulin Z. (2009). Study and Application of Gelled Foam for In-Depth Water Shutoff in a Fractured Oil Reservoir. J. Can. Pet. Technol..

[5] He J., Ye Z., Liao D., Chen Q.M., Liu H.R., Fan H.B., Luo M., Zhong C. (2024). Synthesis of a water-soluble high hydroxymethyl content phenolic resin crosslinker and the associated polyacrylamide weak gel property investigation. J. Polym. Sci..

[6] Song T., Bai B., Eriyagama Y., Schuman T. (2023). Lysine Crosslinked Polyacrylamide—A Novel Green Polymer Gel for Preferential Flow Control. ACS Appl. Mater. Interfaces.

[7] Nebhani L., Choudhary V., Adler H.J.P., Kuckling D. (2016). pH- and Metal Ion- Sensitive Hydrogels based on N-[2-(dimethylaminoethyl)acrylamide]. Polymers.

[8] Baiyu Z., Hongming T., Senlin Y., Gongyang C., Feng Z., Ling L. (2022). Experimental and numerical investigations of particle plugging in fracture-vuggy reservoir: A case study. J. Pet. Sci. Eng..

[9] Xu H., Zhang L., Wang J., Jiang H. (2023). Evaluation of Self-Degradation and Plugging Performance of Temperature-Controlled Degradable Polymer Temporary Plugging Agent. Polymers.

[10] Carpenter C. (2022). Study Investigates Foam Use for Control of Pipeline Slugging. J. Pet. Technol..

[11] Hao F., Liu K. (2024). Introduction for the special issue on deep petroleum systems. AAPG Bull..

[12] Tian J., Liu J., Liu S., Yuan S., Cai W., Jiang H. (2023). A gel system with long-term high-temperature stability for deep profile modification of high-temperature oil and gas reservoirs. J. Appl. Polym. Sci..

[13] Xu P., Yu J., Xie L. (2024). Synthesis and Evaluation of Plugging Gel Resistant to 140 °C for Lost Circulation Control: Effective Reduction in Leakage Rate in Drilling Process. Polymers.

[14] Golcuk S., Muftuoglu A.E., Celik S.U., Bozkurt A. (2013). Synthesis and characterization of polymer electrolyte membranes based on PVDF and styrene via photoinduced grafting. J. Polym. Res..

[15] Liu C., Fu L., Jiang T., Liang Y., Wei Y. (2021). High-strength and self-healable poly (acrylic acid)/chitosan hydrogel with organic-inorganic hydrogen bonding networks. Polymer.

[16] Ilavya A., Rathwa P., Paine S., Makwana M., Bera A. (2025). Gelation studies of nanographene oxide-augmented nanocomposite polymer gel systems for water shutoff technique in oil reservoirs. J. Mol. Liq..

[17] Liu H., Li X., Pan Z., Dai L., Zhang M., Shen F., Si C. (2025). Lignin-based plugging hydrogel with high-temperature resistance and adjustable gelation. Adv. Compos. Hybrid Mater..

[18] Bai X., Wang M., Chen Y., Wu L., Yu J., Luo Y. (2024). Synthesis and properties of self-healing hydrogel plugging agent. J. Appl. Polym. Sci..

[19] Zhu D., Hou J., Meng X., Zheng Z., Wei Q., Chen Y., Bai B. (2017). Effect of Different Phenolic Compounds on Performance of Organically Cross-Linked Terpolymer Gel Systems at Extremely High Temperatures. Energy Fuels.

[20] Wang J., Lou L., Qiu J. (2019). Super-tough hydrogels using ionically crosslinked networks. J. Appl. Polym. Sci..

[21] Ye D., Chang C., Zhang L. (2019). High-Strength and Tough Cellulose Hydrogels Chemically Dual Cross-Linked by Using Low- and High-Molecular-Weight Cross-Linkers. Biomacromolecules.

[22] Li X., Fu M., Hu J. (2024). Preparation and Performance Evaluation of Temperature-Resistant and Salt-Resistant Gels. Gels.

[23] Lu S., Liu Q., Li P., Zhao G., Xu B., Li J., Dai C. (2024). Preparation and enhancement mechanisms of a novel modified nanographite hybrid polymer gel for profile control in deep reservoirs. Colloids Surf. A Physicochem. Eng. Asp..

[24] Wei Z., Zhang J., Liu J., Yang L., Zhang L., Wang J., Fan H. (2025). Enhancing thermal stability of preformed particle gels (PPGs) under high temperature: The role of crosslinkers. J. Mol. Liq..

[25] Zhang J., Li B., Xin Y., Li B., Zhang M., Wang H., Zhang S., Zhang H., Gu X. (2024). Preparation and characterization of high-stability gel foam for fracture plugging in reservoirs. Phys. Fluids.

[26] Chen X.W., Wang T.G., Yin W.J., Zhang L.S., Sun S.D. (2024). All-natural plant-based HIPE-gels simultaneously stabilizing with Quillaja saponin and soy protein isolate: Influence of environmental stresses on stability. Food Hydrocoll..

[27] Yang H., Iqbal M.W., Lashari Z.A., Cao C., Tang X., Kang W. (2019). Experimental research on amphiphilic polymer/organic chromium gel for high salinity reservoirs. Colloids Surf. A Physicochem. Eng. Asp..

[28] He Y., Guo J., Bai J., Hua L., Zhang Y., Huang Z., Pan L., Hong Z. (2024). An Innovative High-Strength Double-Network Hydrogel for Use as a Drilling Fluid Plugging Agent. Gels.

[29] Guo H., Ge J., Li L., Liu M., Wang W. (2024). Development, evaluation and stability mechanism of high-strength gels in high-temperature and high-salinity reservoirs. J. Mol. Liq..

[30] Guo H., Ge J., Xu Y., Lv Q., Li Z., Zhou D., Tao Z. (2022). Preparation and mechanism of stability for high-temperature and high-salinity gels. SPE J..

[31] Liu Y., Zhang J., Wu X., Kang X., Guan B., Li X., Ye Y., Xiao P., Wang X., Li S. (2021). Experimental Investigation on a Novel Particle Polymer for Enhanced Oil Recovery in High Temperature and High Salinity Reservoirs. J. Chem..

[32] Sun Z., Wang S., Zhu Q., Cao X., Lv K., Feng Y., Yin H. (2023). Insights into Polyacrylamide Hydrogels Used for Oil and Gas Exploration: Gelation Time, Gel Strength, and Adhesion Strength. Energy Fuels.

[33] Liu Z., Xu J., Peng W., Yu X., Chen J. (2023). The Development and Deployment of Degradable Temporary Plugging Material for Ultra-Deepwater Wells. Processes.

[34] Li H., Liu H., He Z., Li Z., Zhang S., Li Q. (2019). Use Gel to Control Severe Mud Losses in Carbonate Reservoir Formations in Tahe Oilfield. Drill. Fluid Complet. Fluid.

[35] Yang Y., He X., Sun D., Zhang H., Zhong Y., She J. (2023). Pseudointerpenetrating network nanocomposite hydrogel for temporary plugging in fractured reservoirs. Colloids Surf. A Physicochem. Eng. Asp..

[36] El-Karsani K.S., Al-Muntasheri G.A., Sultan A.S., Hussein I.A. (2015). Gelation of a Water-Shutoff Gel at High Pressure and High Temperature: Rheological Investigation. SPE J..

